# Pharmacokinetics and Pharmacodynamics of Immediate- and Modified-Release Mycophenolic Acid Preparations in Healthy Beagle Dogs

**DOI:** 10.3389/fvets.2020.611404

**Published:** 2021-01-28

**Authors:** Michael Klotsman, Sebastien Coquery, Gayatri Sathyan, Vatsala Naageshwaran, Paddy Shivanand, Amanda J. Fairchild, Oliver A. Garden, Wayne H. Anderson

**Affiliations:** ^1^Okava Pharmaceuticals, San Francisco, CA, United States; ^2^WORK-FLOW, Elmsford, NY, United States; ^3^Absorption Systems LP, Exton, PA, United States; ^4^Department of Psychology, University of South Carolina, Columbia, SC, United States; ^5^Clinical Sciences and Advanced Medicine, University of Pennsylvania School of Veterinary Medicine, Philadelphia, PA, United States; ^6^Pulmonary and Critical Care Medicine, University of North Carolina at Chapel Hill, Chapel Hill, NC, United States

**Keywords:** mycophenolic acid, immunomodiilation, pharmacodynamic, canine (dog), immune mediated haemolytic anemia, steroid sparing agents, atopic dermatitis

## Abstract

**Background:** Mycophenolic acid (MPA) is a broad-acting immunomodulating agent that may be therapeutically beneficial for the treatment of immune-mediated diseases in canine patients.

**Objectives:** To determine the suppressive effects of MPA on T-cell proliferation, and to assess the feasibility of a canine-specific q24 h modified-release MPA formulation (OKV-1001b).

**Animals:** Fifteen healthy purpose-bred male beagle dogs.

**Methods:** Two nearly identical open-label fifteen-day studies were conducted in which dogs were randomized to receive mycophenolate mofetil (MMF; 10 mg/kg q12h), or two doses of OKV-1001b (270 mg and 180 mg; q24h). Serial pharmacokinetic (PK) and pharmacodynamic (PD) samples were collected on Days 1, 8, and 15. MPA plasma concentrations were measured by liquid chromatography-tandem mass spectrometry (LC-MS/MS), while an *ex vivo* T-cell proliferation assay assessed PD effects. Dogs were continuously monitored for evidence of side effects and gastrointestinal tolerability.

**Results:** MPA induced inhibition of T-cell proliferation was observed following administration of all MPA preparations in a clear concentration-dependent manner. The PK/PD relationship was maintained across all days and time-points. Data generated herein suggest that MPA plasma concentrations above 600 ng/mL achieve at least 50% inhibition of T-cell proliferation.

**Conclusions and Clinical Importance:** MPA holds therapeutic potential for treating dogs with immune-mediated disease, but clinical trials will be necessary to determine its safety and efficacy in naturally occurring disease. Likewise, q24h oral modified release MPA preparations that maintain MPA plasma concentrations between 600 and 1,000 ng/mL are warranted for further studies in client-owned dogs.

## Introduction

Mycophenolic acid (MPA), a broad-acting immunomodulating agent, is commonly used in human medicine to treat patients with immune-mediated inflammatory diseases (1, 2). There is growing evidence that the immunomodulating properties of MPA are therapeutically beneficial for the treatment of immune-mediated diseases in canine patients (3, 4). The immediate-release (IR) MPA prodrug mycophenolate mofetil (MMF) is available as a generic drug and is the most common MPA preparation administered to dogs (4, 5). Despite common use in humans, target MPA plasma concentrations and dosing regimens are poorly defined in canines.

In human medicine, target MPA exposure is not always achieved with standardized dosing. Five to ten-fold variations in dose-normalized MPA exposure have been reported (2, 6, 7); this variability places patients at risk of under-exposure and lack of efficacy or over-exposure and toxicity (8). The intricate balance between under- and over-immunomodulation becomes increasingly complex in canine patients. Reliance on human MPA dosing protocols can be misleading because factors such breed-specific differences (e.g., size) and other properties of canine physiology can uniquely impact the pharmacokinetics and absorption characteristics of orally administered drugs (9, 10). Moreover, MPA pharmacokinetic (PK) and pharmacodynamic (PD) relationships may differ markedly across different species (11, 12).

Modified release (MR) oral dosage forms designed to improve the performance of IR formulations [e.g., increase the therapeutic activity compared to the intensity of side effects, reduce the number of drug administrations required during treatment (13)] are commonly developed for human applications (14, 15). For example, an enteric coated formulation designed to improve the gastrointestinal (GI) tolerability of MPA has been developed (16, 17). Although the dog is often used as a preclinical species during human drug formulation development (18), advanced MR drug delivery technologies have not been widely adopted for orally administered companion animal products (19, 20). A modified release multiparticulate MPA formulation, under development for dogs, is designed to achieve the following goals: (1) to improve dosing compliance by extending the duration of therapeutically effective drug concentrations (e.g., q24 h); (2) to mitigate GI side effects by delaying drug release and by blunting peak plasma concentrations (C_max_) in order to minimize concentration-dependent side effects; and (3) to provide a slow rate of drug release in order to minimize large intra- and inter-patient fluctuations in PK.

The objectives of this study were two-fold. First, we sought to gain additional insight into MPA dosing requirements for dogs. To achieve this objective, an *ex vivo* whole blood T-cell proliferation assay that is well suited for characterizing the suppressive effects of MPA (12, 21, 22) was used to establish a reference PD time course profile in healthy beagle dogs (23, 24). MPA plasma concentrations relative to PD effects following single- and repeated-dosing of MMF were evaluated. Obtaining more clarity into the complex interplay of the MPA plasma concentration and lymphocyte proliferation (i.e., the PK/PD relationship) in a canine model is an important step (24, 25) that will help to establish dosing regimens and standardize treatment protocols for dogs presenting with immune-mediated diseases. The second objective of this study was to concept-test a prototype MR MPA formulation (OKV-1001b). To this end, PD effects of OKV-1001b were evaluated and contrasted to that of IR MMF following single- and repeated- oral administration. These data will help to assess the therapeutic potential of a canine-specific MR formulation, and help to define formulation requirements needed to improve the clinical performance (e.g., PK profile) of a MR MPA formulation tailored to the unique needs of dogs.

## Materials and Methods

### Study Design

**Figure 1:**
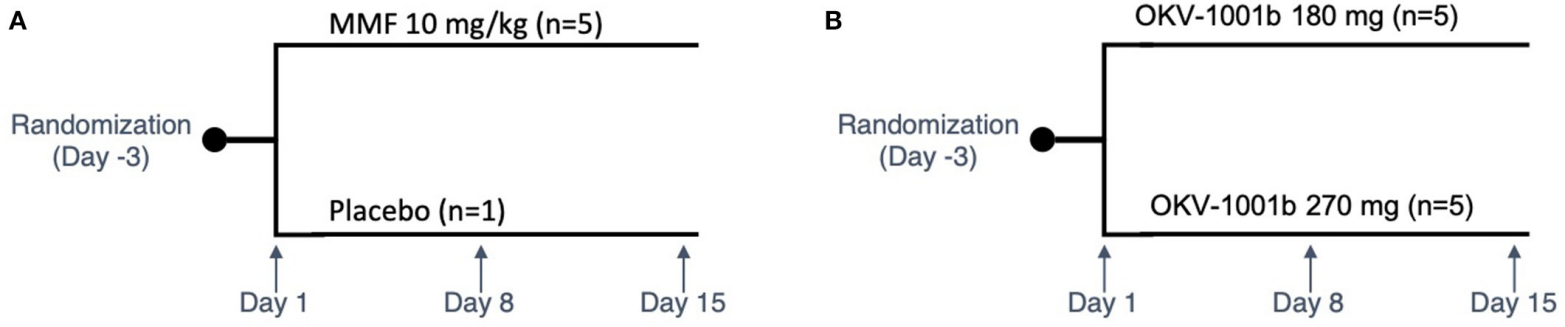
Study design schema. Study 1 is shown in **(A)**. In Study 2 **(B)**, two groups of dogs were orally administered OKV-1001b once-daily for a 15-day period. PK and PD samples were collected on Days 1, 8, and 15. On each of these days, samples were collected at pre-dose, and at 2.5, 4, and 8 h post-dose. On Day 8, additional PK samples were collected at 1, 2, and 6 h mark.

Purpose-bred male beagle dogs (Marshall BioResources, North Rose, NY, USA) were studied in two nearly identical open-label fifteen-day PK/PD studies (Figure 1). At the time of enrollment, the dogs (*N* = 15) were at least 2 years of age (range: 2.9–5.3 years), with weights ranging from 9.2 to 15 kg. In Study 1 (IACUC protocol #18C341Q1), a group of dogs (*n* = 5) were randomized to receive MMF (10 mg/kg PO q12h). In Study 2 (IACUC protocol #18C341Q2), two groups of dogs (*n* = 5 each) were randomized to receive two different doses of OKV-1001b (270 and 180 mg PO q24h). Both studies also included an additional healthy control dog that was sampled on Days 1, 8, and 15.

The dogs were considered healthy at time of enrollment based on physical examination. The general health of each animal was assessed throughout the study. The feces of all animals were observed for evidence of GI side effects and recorded at least twice daily over the active study duration, grading fecal quality using a modified WALTHAM® feces scoring system (Supplementary Table 1) (26).

Three days prior to the start of the study, the dogs were acclimated to a once daily morning feeding schedule consisting of 500 g 1:1 mixture of a dry certified laboratory diet (5,006 laboratory canine diet from Lab Diet) and canned wet dog food (Alpo). Dogs were fed at approximately the same time each morning, and all food was removed 1 h prior to administration of the first daily dose. The quantitative amount of food provided and consumed by each animal was recorded throughout the study. Water was supplied *ad libitum* to the animals throughout the study.

The study was conducted at an AAALAC accredited and USDA registered testing facility. During the study, dogs were housed in individual kennels in compliance with current recommendations for the Guide for the Care and Use of Laboratory Animals and under the standard operating procedures of the testing facility. The study protocols were approved by the test facility Institutional Animal Care and Use Committee (IACUC).

### Drug Administration

MMF oral suspension was prepared according to the manufacturer's instructions (MMF, CellCept® Genentech, South San Francisco). Each day, a stock concentration of 200 mg/mL MMF solution was used to create a 10 mg/mL dosing solution. The dosing solution was titrated to 10 mg/kg (or 1 mL for each kg in dog weight), and then administered via rubber oral gavage tube that was subsequently flushed with ~10 mL of water. Based on the available published literature (3, 4), 10 mg/kg PO q12h administration of MMF (equivalent to 7.39 mg/kg MPA) was selected as an appropriate starting dose for dogs. Each dog was dosed in the morning (time _0_) and 12 h later in the evening for 14 days (Days 1–14). MMF was only administered in the morning of Day 15. Doses were given at approximately the same time of day on all days.

The experimental OKV-1001b formulation was generated by coating an aqueous solution of Na mycophenolate (MPS) on commercially sourced microcrystalline cellulose spheres, which were further coated with a release rate-controlling membrane and an enteric coat. The release rate-controlling membrane was designed to slow and extend the *in vivo* release of MPA for an extended duration, while the enteric coat was designed to delay release until the beads exited the stomach. The experimental drug product, consisting of a capsule filled with MPS beads, was manufactured, tested and released for use in animal studies by Emerson Resources, Inc. (600 Markley St. Norristown, PA 19401).

Based on prior PK assessments (unpublished data submitted to FDA), a 270 mg dose (OKV-1001b_270_) was selected for initial testing of q24h PO dosing of OKV-1001b (i.e., equivalent to 256 mg total daily MPA). A secondary 180 mg (i.e., equivalent to 165 mg total daily MPA) q24h OKV-1001b dose (OKV-1001b_180_) was also tested. Capsules were orally administered, followed by gentle flushing with 10 ml of water.

### Pharmacokinetic Blood Sampling and Assay

On Days 1, 8, and 15, 2 mL serial blood samples were collected via the jugular vein prior to dosing (pre-dose) and at 45 min, 4 h, and 8 h after MMF administration. For OKV-1001b, Day 1, Day 8, and Day 15 sampling occurred at pre-dose, and 2.5, 4, and 8 h after administration. Additional Day 8 PK samples for OKV-1001b were taken at 1, 2, and 6 h.

The aliquots of collected whole blood were placed in chilled polypropylene tubes containing K_2_EDTA. Samples were maintained chilled throughout processing. Blood samples were centrifuged at 3,000 g for 5 min at 4°C. The resulting plasma was transferred to polypropylene tubes containing 10% formic acid to create a 10:1 ratio of plasma to formic acid. The samples were then mixed, placed on dry ice, and stored in a freezer maintained at −80°C until analyzed.

A liquid chromatography (LC)-tandem mass spectrometry (MS)/MS method for the determination of MPA in canine plasma was developed and qualified according the Guidelines for Bioanalytical Method Validation published by the Food and Drug Administration (FDA) (Supplementary Material). The specificity, accuracy, and precision of the method were evaluated in triplicate using 50 μL aliquots of acidified beagle dog plasma, precipitated with 150 μL of acetonitrile containing the internal standards MPA-d_3_ and AcMPAG-d_3_ (Toronto Research Chemicals, North York, Ontario, Canada). MPAG-d_3_ was obtained from Cerilliant (Round Rock Texas). MPA and MPAG were obtained from Santa Cruz Biotechnology (Santa Cruz California) and AcMPAG from Toronto Research Chemicals. Stock solutions were prepared in DMSO (1 mg/ml) and stored at −2–8°C. The calibration curve consisted of eight standards ranging from 1.00 to 1,000 ng/mL for each analyte. Quality control (QC) samples were prepared at the lower limit of quantification (LLOQ 1.00 ng/mL), Low QC (3.00 ng/mL), Mid QC (75.0 ng/mL), and High QC (750 ng/mL) concentrations. Calibration standards and QC samples were prepared from independent stock solutions. After vortexing and centrifugation of the calibration curve samples, the resulting supernatant was transferred to a clean 96-well plate and capped for analysis. Chromatography was performed on a Waters Acquity UPLC HSS T3, 2.1 × 50 mm column and analyzed by electrospray MS in the positive ion mode. A gradient program was used to elute the analytes using 4 mM ammonium formate as mobile phase A and 4 mM ammonium formate in 90:10 acetonitrile:water as mobile phase B. With a flow rate of 0.300 mL/min, each analyte and their respective internal standards co-eluted with retention times of ~2.72 min for MPA /MPA-d_3_. Total run time was ~4.25 min. Using a 50 μL plasma sample volume, the range of the assay was 1.00–1,000 ng/mL for each analyte. Accuracy of the calibration curve standards to within ±15% (±20% at LLOQ) of the nominal concentration was achieved. Additional qualification assessments included bench-top stability at room temperature, freeze/thaw stability, long-term freezer stability at −20°C, and stock solution stability. Assays were conducted under Good Laboratory Practices (GLP) [FDA 21 Code of Federal Regulations (CFR) Part 58] at an independent laboratory (Absorption Systems, 436 Creamery Way, Exton, PA 19341).

### Pharmacodynamic Blood Sampling and Assay

PD (4 mL whole blood) samples were collected *via* the jugular vein at four sampling time-points. On Days 1, 8, and 15, samples were collected at pre-dose, 45 min for MMF (2 h for OKV-1001b), 4 and 8 h post-dose. The pre-dose samples on Day 1 were used to calculate baseline.

T-cell proliferation was evaluated using a previously described flow cytometry assay (11, 27, 28) that was further modified as described below. Heparinized whole blood samples were diluted 1:4 in cRPMI (Roswell Park Memorial Institute 1640 + L-glutamine + Pen/strep). One hundred microliters of diluted whole blood were plated in 96-well plates. Cells were incubated with 100 uL of Concanavalin A (ConA Sigma) at 10 μg/mL or cRPMI for 72 h at 37°C and 5% CO_2_. The concentration of ConA and incubation times were adjusted to provide optimal proliferation and cell viability.

Following incubation, erythrocytes were lysed with multi-species lysis buffer and centrifuged. Leukocytes were stained with eBioscience™ Fixable Viability Dye eFluor 450 (Thermo Fisher Scientific 65-0863-14), washed, spun, and subsequently surface stained with Rat Anti-Dog CD5 APC (Bio-Rad MCA1037APC, clone YKIX322.3) and Rat anti-Dog CD45 RPE (Bio-Rad MCA1037APC, clone YKIX716.13). Cells were fixed/permeabilized (eBiosciences 00-5123-43 Fixation/Permiabilization Buffer) and incubated with Mouse anti-Human/Dog Ki-67 FITC (Thermo Fisher Scientific 11-5698-80, clone SolA15). Cells were washed, spun and re-suspended in flow cytometry staining buffer for analysis. Compensation controls used OneComp eBeads™ Compensation Beads (Thermo Fisher Scientific 01-1111-41) for the surface and intracellular antibodies, and cells for the live/dead marker.

Samples were acquired on an Attune NxT flow cytometer (Thermo Fisher Scientific) at a validated speed of 100 μL/min; at least 200,000 gated events were collected. The following gating strategy was used: Scatter (FSC/SSC) > Singlets (FSC-A/FSC-H) > Live (L-D/SSC) > T cells (CD45+/CD5+) > Proliferating (CD5+/Ki-67+) (Supplementary Figure 1).

Generated Flow Cytometry Standard (FCS) files were analyzed with FlowJo v 10.4.2 (BD Biosciences). The percent of proliferating cells after stimulation was calculated by subtracting the percent CD5+/Ki-67+ cells in medium alone (unstimulated) from the percent CD5+/Ki-67+ cells stimulated with mitogens. Data were reported as percent of baseline calculated at each time-point after OKV-1001b administration as: Percent of Baseline (POB) = [1-(treatment/pretreatment)] × 100] where pre-treatment refers to results obtained from stimulated blood without MPA at the Day 1 pre-dose, and treatment refers to stimulated samples collected at all subsequent time-points.

### Pharmacokinetic Analysis

Using the OKV-1001b samples collected on Day 8, standard non-compartmental pharmacokinetic parameters (C_max_, T_max_, and AUC_8_) were estimated using Phoenix Winnonlin software 64 (Build 7.0.0.2535) for MPA. Nominal (protocol) times were used for the analysis. The area under the concentration-time curve from zero to 8 h post-dose was calculated using the linear up/linear down trapezoidal method. To explore dose-proportionality, a lower 180 mg q24h OKV-1001b dose, designated as Dose 2 (OKV-1001b_180_), was selected.

### Statistical Analysis

MMF (10 mg/kg q12h) was used to establish a reference PD time course. Based on prior PK assessments (unpublished data), the 270 mg OKV-1001b dose was *a priori* selected as the comparator to MMF.

A three-way, mixed effect analysis of variance (ANOVA) was run to evaluate the impact of drug (i.e., MMF and OKV-1001b), day, and timepoint on lymphocyte suppression (as measured by proportion change from baseline). All main effects and interactions among the predictor variables were estimated in a fully crossed 2 × 3 × 4 factorial model (drug: two-level, between dogs factor; day: three-level, within dogs factor; and timepoint: four-level, within dogs factor), with dog modeled as a random effect. Follow-up analyses were conducted as appropriate to statistically probe significant effects in the model (29). Specifically, we analyzed a simple two-way interaction of day × timepoint for each level of drug. Primary analyses were run using the lme4 package in R version 4.0.0 (“Arbor Day”), where model parameters were estimated using restricted information maximum likelihood and compared to a nominal alpha criterion of *p* < 0.05. For all model estimates, the applicable degrees of freedom, *F*-value, and *p*-value are reported. Follow up tests were conducted in conventional general linear modeling (GLM) and paired *t*-test frameworks, adjusting inferential tests for multiple comparisons (30). Partial eta-squared estimates were calculated for GLM model estimates to demonstrate the proportion of variance explained uniquely by given model parameter estimates, and Cohen's d values were calculated for paired *t*-test estimates to demonstrate the magnitude of given mean differences.

Pearson's r was used to assess the correlation between lymphocyte suppression and MPA concentration measurements. Significance was set at *p* ≤ 0.05. An inhibitory sigmoid E_max_ model was used to describe the relationship between the concentration of MPA and drug effect (i.e., POB) according to the following equation:

$$\begin{array}{l} \text{Y}=\text{Min}+\;\frac{\text{Max}-\text{Min}}{1+{\left(\frac{\text{X}}{\text{E}{\text{D}}_{50}}\right)}^{\text{Hill coefficient}}} \end{array}$$

where Y is the drug effect, Min is the effect at zero drug concentration (baseline), Max is the maximum effect, X is the plasma MPA concentration, ED_50_ the MPA concentration required for 50% effect-inhibition (i.e., the MPA concentration needed to obtain 50% of the E_max_–this concentration is also called the 50% inhibitory concentration, IC_50_), and the Hill coefficient (i.e., sigmoidicity constant) (31).

Modified WALTHAM® feces scores were summarized descriptively. Dogs that did not defecate were assigned a score of zero, while an average daily score was used if more than one time-point was available.

## Results

### Descriptive Statistics and MPA Pharmacokinetics

The 180 mg and 270 mg doses of OKV-1001b corresponded to an MPA dose of 11.9–17.1 mg/kg and 18.0–28.8 mg/kg, respectively. Post-dose PD samples for MMF dog #5 on Day 1 were discarded due to a processing error.

Following administration of MMF, mean MPA concentrations increased rapidly with high plasma concentrations observed at the 45-min timepoint (Supplementary Table 2). At the 4- and 8-h timepoints, mean MPA concentrations declined by over 80% relative to the peak concentrations observed at the 45-min timepoint (Supplementary Table 2). Mean MPA concentrations were highly variable. Variability was most pronounced at the 45-min timepoint on Day 1 (range: 380–5,040 ng/mL), Day 8 (range: 1,580–9,030 ng/mL), and Day 15 (range: 152–4,650 ng/mL).

**Figure 2:**
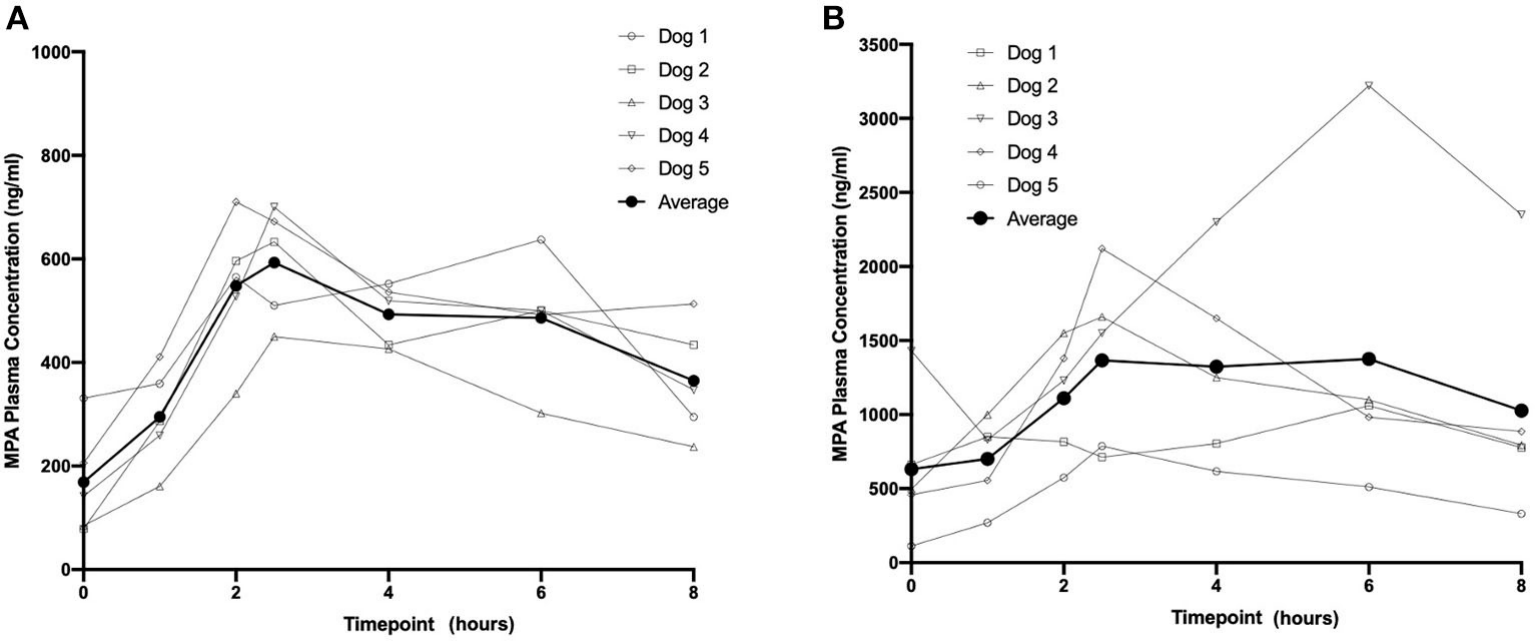
Eight-hour pharmacokinetic profile of OKV-1001b 180 mg **(A)** and 270 mg **(B)** measured following 8 days of drug administration.

Based on visual inspection of the data and comparisons of concentrations on Day 8 vs. Day 15 at corresponding timepoints, steady state appears to have been attained by Day 8 for all three dosing regimens (data not shown). The average steady-state MPA concentration (Table 1) vs. time profiles were relatively flat after administration of the two OKV-1001b doses (Figure 2). The highest average concentrations occur around 2.5 h post-dose and are roughly 3–4 times higher than the trough values. There is higher variability at the 270 mg dose compared to the 180 mg dose. Exposure of MPA increased in a slightly greater than dose proportional manner from 180 to 270 mg. Variability was quite low (<20%) in the 180 mg dose group but higher (>50%) in the 270 mg dose group.

**Table 1:** Steady-state (day 8) PK of 180 mg and 270 mg OKV-1001b in healthy beagle dogs.

**OKV-1001b dose (mg)**	**Cmax (ng/mL)**	**Tmax (h)**	AUC8 (ng*h/ml)
180 mg	626 ± 104	2.5 (2.5–6)	3,584 ± 649
270 mg	1,769 ± 963	2.5 (2–6)	9,310 ± 4,780

*T_max_ values are displayed as median (range), all other values are mean ± SD*.

### Immediate-Release MMF Pharmacodynamics

**Figure 3:**
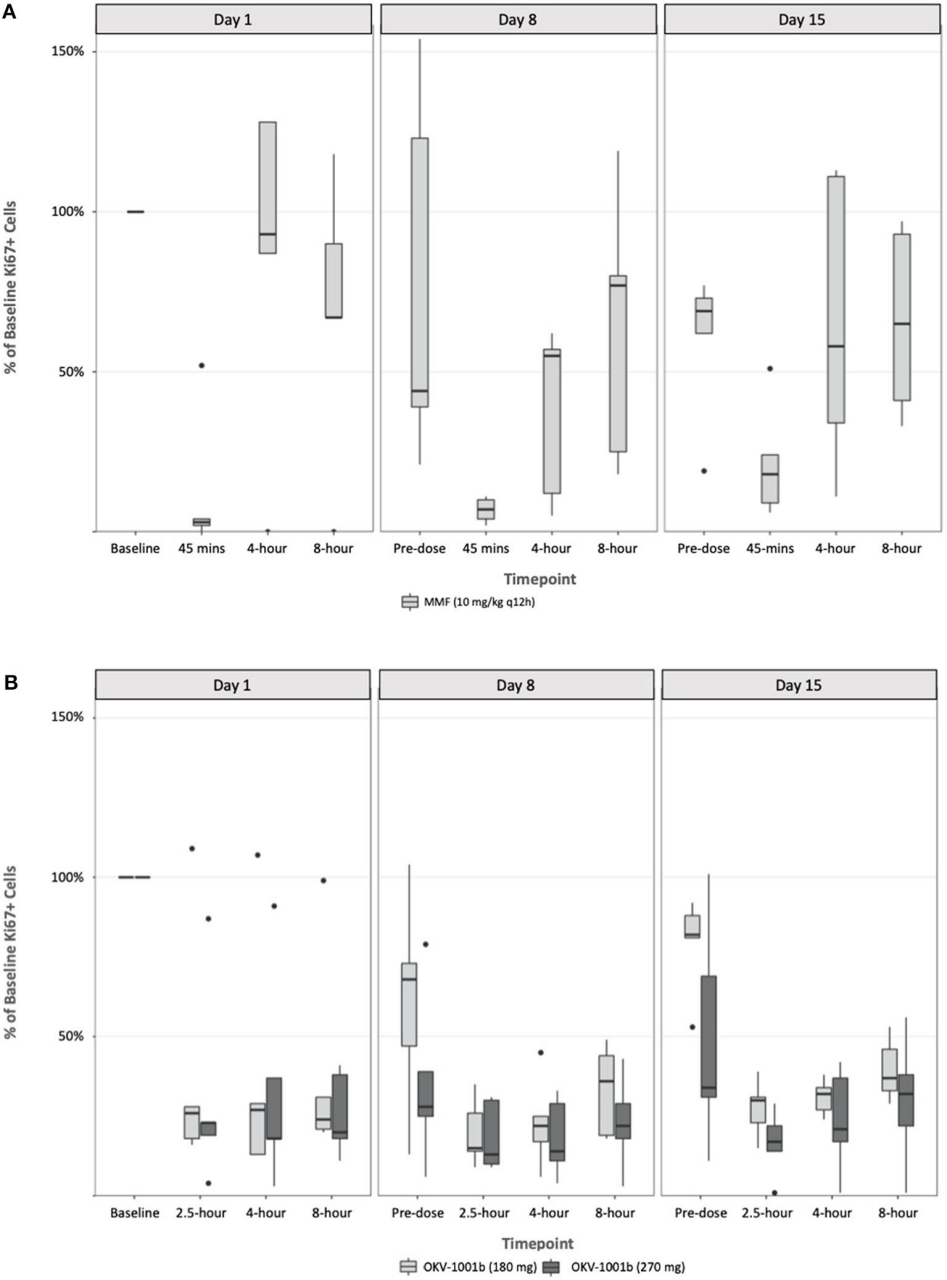
Pharmacodynamics of single- and repeat administration of MMF **(A)** and OKV-1001b (280 mg and 180 mg; **B**), as measured by *ex-vivo* mitogen stimulated T-lymphocyte proliferation. All pharmacodynamic results are expressed as percent inhibitions of pretreatment assay values for each dog. Boxes and horizontal bars denote interquartile range (IQR) and median percent inhibition, respectively. Whisker endpoints are equal to the maximum and minimum values below or above the median ± 1.5 times the IQR.

Following a single administration of MMF, rapid and maximum inhibitory effects were found to coincide with peak MPA plasma concentrations (Figure 3). At higher MPA plasma concentrations observed at the 45-min timepoint, a >80% decrease in the percentage of proliferating CD45+ CD5+ cells was observed. The percentage of proliferating cells returned to baseline at the 4-h (median = 111%; range 87–128%) and 8-h timepoints (median = 79%; range 67–118%) (Figure 3). The percentage of proliferating cells remained stable and did not decrease in the control dog (data not shown).

Following seven continuous days of q12h dosing with MMF, the pre-dose percentage of proliferating CD45+ CD5+ were suppressed by a median of 17% (range 21–154% of baseline; Figure 3). Similar to what was observed on Day 1, a significant decrease in the percent of Ki-67+ cells relative to baseline was observed at the 45 min timepoint on Days 8 and 15. On Day 8, the median percent of baseline proliferating lymphocytes was 55% (range: 5–62%) and 77% (range: 18–119%) at 4- and 8-h, respectively. A similar pattern was observed on Day 15 with highly variable PD responses observed at the 4- and 8-h timepoints (Figure 3).

### Modified-Release OKV-1001b Pharmacodynamics

Following a single administration of OKV-1001b_270_, a >75% decrease in the percentage of proliferating CD45+ CD5+ cells was observed at the 2.5 h, and maintained at the 4-, and 8-h timepoints (Figure 3). Significant inhibition (~70%) was observed at the pre-dose timepoints on Days 8 and 15. OKV-1001b_270_ PD effects were subsequently measured on Day 8 and Day 15. Consistent with the day 1 single-dose observations, evidence of MPA's inhibitory effect on T-cell proliferation was observed across all timepoints on Days 8 and 15 (Figure 3). Similar inhibitory effects were observed for OKV-1001b_180_ (Figure 3).

Box plots of the distributions of the percentage of proliferating CD45+ CD5+ suppressed by MMF and OKV-1001b_270_ show overall group mean differences (Figure 3). A three-way mixed effects ANOVA revealed a statistically significant three-way interaction among drug × day × timepoint on lymphocyte suppression [F_(6, 85)_ = 2.719, *p* = 0.018], indicating that the drug effect was conditional on both day and timepoint.

Follow-up tests were performed to probe how day and timepoint influenced drug response across the MMF and OKV-1001b groups. A significant day × timepoint interaction was found for the MMF group [F_(6, 45)_ = 4.862, *p* < 0.001], but not for the OKV-1001b group [F_(6, 48)_ = 2.529, *p* = 0.033], after controlling for multiple comparisons (i.e., α= 0.05 divided by two comparisons yields α=0.025). Likewise, there was neither a main effect of day [F_(2, 48)_ = 0.635, *p* = 0.534] nor timepoint [F_(3, 48)_ = 0.203, *p* = 0.894] in the OKV-1001b group, thus demonstrating that, in contrast to what was observed for the MMF formulation, drug response to OKV-1001b was stable across day and time.

To better understand the interactive effect that day and timepoint had on drug response in the MMF group, we analyzed the simple main effects of timepoint within each day. Results indicated that, after adjusting for multiple comparisons, the timepoint effect was statistically significant on Day 1 (*p* < 0.001), Day 8 (*p* = 0.038), and Day 15 (*p* = 0.38). The magnitude of effect for timepoint was greatest on Day 1 (n2 = 0.87), with large effect sizes also observed on Day 8 (n2 = 0.372) and Day 15 (n2 = 0.307). These results demonstrate greater variability following single-dose administration on Day 1, with less variability once steady-state PK was reached on Day 8 and Day 15. In particular, the percentage of proliferating cells that rebounded to baseline levels at the 4- and 8-h timepoints on Day 1 were greater than observed on Days 8 and 15 (Figure 3).

To follow up the main effect of timepoint on Day 1 in the MMF group, simple simple comparisons were conducted to investigate pairwise differences among timepoints within Day 1. Results revealed significant pairwise differences between the 45-min timepoint and all other timepoints (all adjusted *p*-values < 0.05, range: *p* = 0.019–0.042), indicating that lymphocyte suppression was achieved at 45 min, but not at the 4- or 8-h timepoints, on Day 1.

### Modified-Release OKV-1001b Pharmacokinetic and Pharmacodynamic Relationship

**Figure 4:**
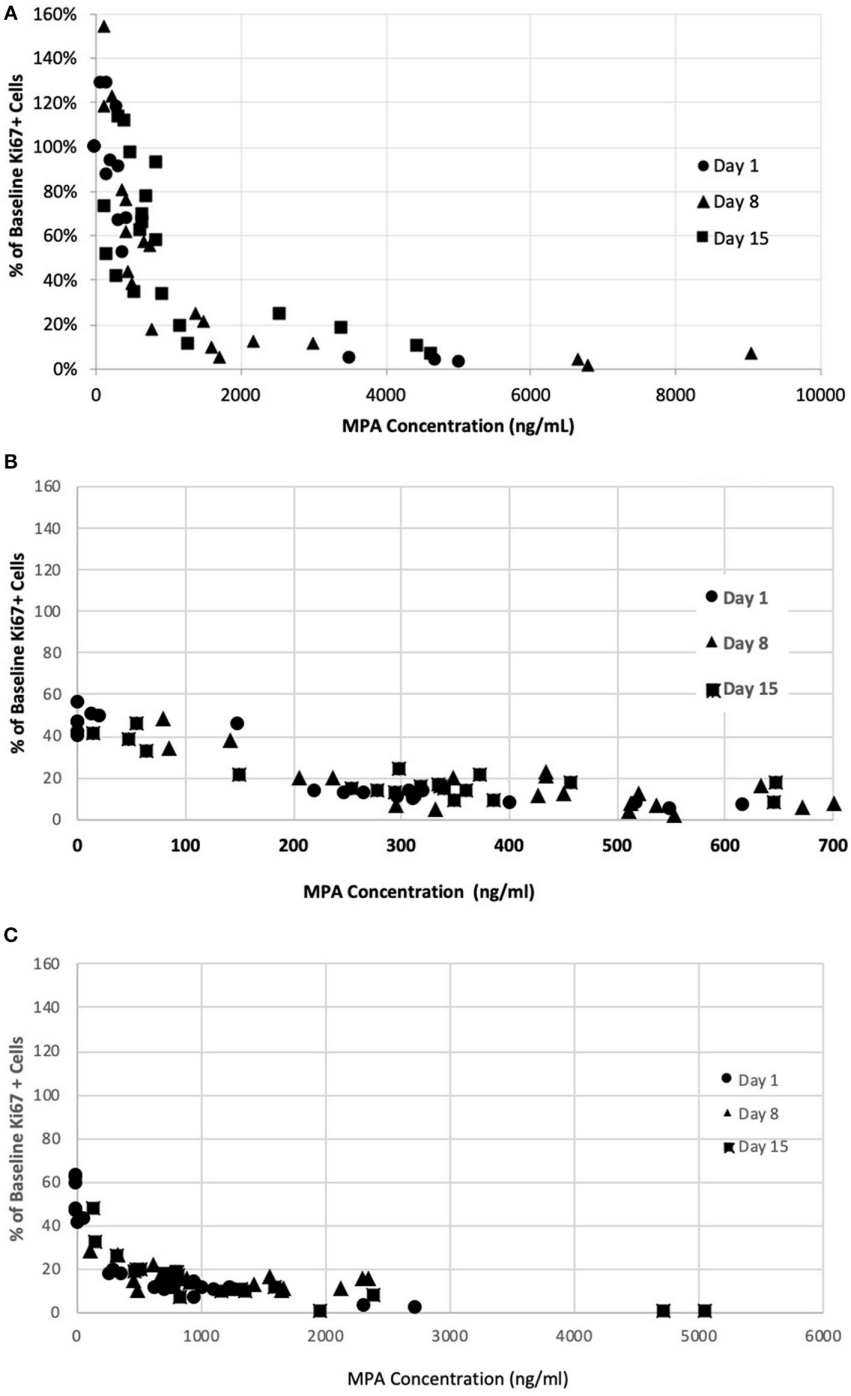
Correlation between MPA plasma concentration in healthy dogs and inhibition of mitogen-stimulated T-lymphocyte proliferation after administration of single- and repeat-doses of MMF **(A)**, OKV-1001b180 **(B)**, OKV-1001b270 **(C)**.

The PK/PD relationship was examined across all timepoints. A consistent concentration-dependent relationship was observed, independent of dose administered, time-point measured, or day of measurement (Figure 4). Plasma MPA concentrations were correlated to percent inhibition for OKV-1001b_270_ [*r* = −0.63 (95% CI = −0.77 – −0.44), *p* < 0.0001] and MMF [*r* = −0.68 (95% CI = −0.80 – −0.51), *p* < 0.0001]. The estimated ED_50_ for OKV-1001b_270_ and MMF were 279 and 672 ng/mL, respectively.

### Safety Observations and GI Events

**Figure 5:**
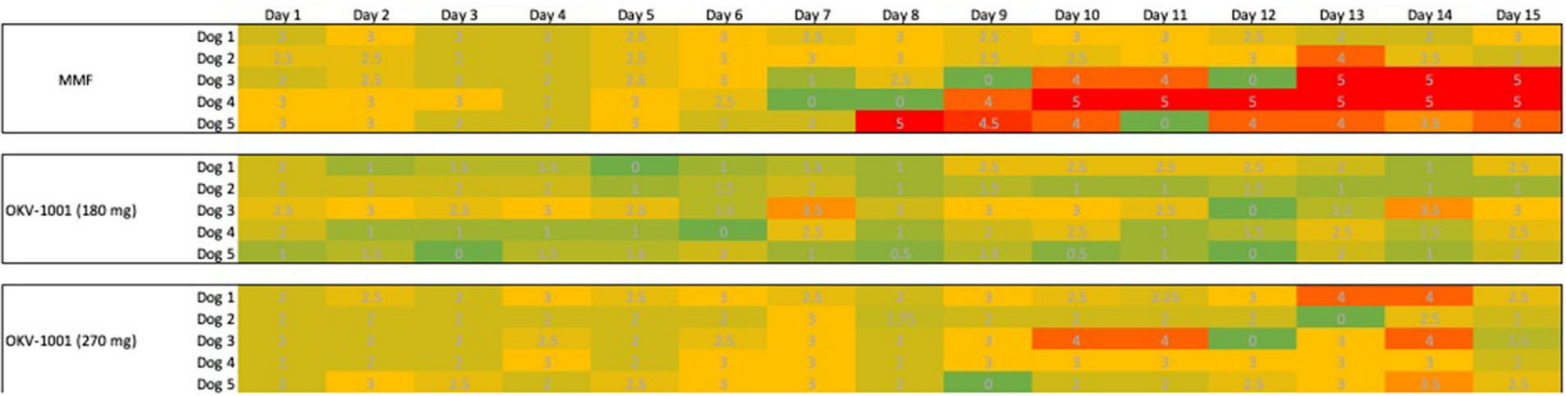
Tabulated heatmap of modified Waltham fecal scores observed in dogs receiving MMF and OKV-1001b 270 mg. Daily scores were coded from low (Green = 0), medium (3 = yellow) to high (5 = Red).

The dogs randomized to MMF and both OKV-1001b doses appeared to tolerate oral administration of study drug, with no dogs prematurely removed from the study. All dogs had normal physical examinations with no visible indicators of adverse effects. No apparent signs of GI intolerance were observed during the first ten days of treatment (Figure 5). In three out of five dogs receiving MMF (10 mg/kg q12h), slightly higher Waltham fecal scores were recorded Days 11 to 15 (Figure 5). One dog in the OKV-1001b_270_ arm had a fecal score of 4 on Days 13 and 14, but no dogs recorded a Waltham score of 5.

## Discussion

MPA, a cornerstone immunomodulator for human transplant patients, is commonly used off-label for the treatment of immune-mediated inflammatory diseases such as lupus (32) and other inflammatory skin diseases (33). Decades of human clinical experience shows that MPA is highly effective (2, 6), but its use is complicated by marked intra- and inter-patient PK variability (2, 6, 7, 34). The use of MMF is increasingly being used to treat dogs with immune-mediated diseases (4, 12), but its clinical utility in veterinary medicine may also be limited by PK variability. Various therapeutic drug monitoring (TDM) strategies are used in human medicine to achieve desired outcomes (7), but there is a notable absence of available TDM methods to aid in determining the dose required to treat dogs with immune-mediated diseases (27).

In the present study, the effects of MPA on canine lymphocyte function were evaluated using an *ex vivo* mitogen-stimulated, T cell proliferation assay. MPA induced inhibition of lymphocyte proliferation was observed following administration of MMF, OKV-1001b_270_ and OKV-1001b_180_ to healthy beagle dogs. The pharmacodynamic assessment showed clear concentration-dependent inhibition of T cell proliferation that was correlated with MPA plasma concentration. The PK/PD relationship was maintained across all days and time-points (Figure 4), suggesting that repeated drug administration did not cause changes in drug absorption, distribution, metabolism, clearance, or desensitization to drug effects. Consistent with previous *in vitro* (12) and *ex vivo* studies (35), the observed PK/PD relationship demonstrates that MPA has high efficacy and potency to inhibit T-cell proliferation in a dose-dependent fashion.

The observed PK/PD relationships offer important insights for the dosing of canine patients with MPA-based therapeutics. The diminished PD signal observed for MMF at the 4 and 8 h post-dose timepoints (Figure 3), coupled with its short half-life (4), suggests that repeated daily administration (e.g., q6h to q12h) may be needed to maintain therapeutic drug concentrations. In comparison, the consistent and durable PD response observed across the measured timepoints in dogs administered OKV-1001b suggests that the MR preparation is beneficial in reducing large fluctuations in the PD response. Moreover, the observed levels of inhibition at pre-dose (i.e. at the 24-h timepoint) on Days 8 and 15 suggest that q24h administration of a MR MPA formulation is attainable. Additional studies testing alternative OKV-1001b configurations, in both fed and fasted dogs, will be needed to identify the MPA release rate that maximizes the PD response.

For human transplant and lupus patients, maintaining MPA AUC_0−12_ of at least 30,000 ng h/mL has been associated with more favorable patient outcomes (1, 2, 36, 37). This translates into an average plasma concentration of 1,250 ng/mL, a level consistent with the reported MPA concentration required for 50% effect-inhibition for T cell proliferation (C_50_ = 1,300 ng/mL) in a study of human kidney transplant patients (35). In the present study, which also used a T-cell proliferation biomarker to characterize drug response, we estimated an ED_50_ of 672 ng/mL for MMF. Our results are similar to a recent report that also used an *ex vivo* lymphocyte proliferation assay to estimate the ED_50_ for MPA in dogs (27). Collectively, these results suggest that therapeutically efficacious MPA plasma concentrations in dogs are similar to those reported in humans. Future dose-ranging studies in client-owned dogs with naturally occurring disease will be needed to better understand therapeutic MPA concentration levels in dogs. It will be important to determine whether, as in various human clinical settings, different MPA dosing regimens will be needed to treat different disease states. For example, in humans, treatment guidelines for transplant (38) and lupus nephritis (1) generally recommend MMF dosed at 1.5 g q12h, while lower maintenance doses (e.g., 750 mg q12h) are used to manage chronic conditions such as atopic dermatitis (39).

Our results show that MPA concentrations that exceed the ED_50_ do not proportionally result in stronger inhibitory effects on proliferating lymphocytes. Importantly, the rather flat PD curve observed at higher MPA plasma concentrations is consistent with human studies which have shown that large differences in C_max_ do not translate into meaningful differences in inhibitory activity (36). This supports the rationale for the development of a modified-release MPA formulation designed to temper the MPA C_max_ and extend the duration of action to maintain therapeutic drug concentrations. A similar result may also be achieved by using lower MMF doses (e.g., 5 mg/kg), administered more frequently (e.g., q6h).

This study used a sensitive T cell proliferation assays that directly measured suppression induced by MPA (11, 12, 21). More specifically, the antigen defined by the monoclonal antibody (mAb) Ki-67, a DNA-binding nuclear protein, is a well-established, widely used cellular proliferation biomarker (40, 41) that is phenotypically well suited for measuring the known inhibitory effect MPA has on proliferating lymphocytes (11, 28). One advantage of this *ex vivo* assay is that the MPA underwent absorption, distribution, and elimination in dogs before the blood was removed for testing, and may therefore be more predictive than *in vitro* assays (12). Utilizing a whole blood assay maintains the blood concentration during the stimulation period. A potential limitation of the study is that only one mitogen, Con-A, was utilized, which may or may not recapitulate activation in different disease states.

The inhibitory effect MPA has on proliferating lymphocytes is clear; however, the cellular alterations and systemic inflammatory pathways responsible for the successful actions of MPA remain unclear. In the present study, the systemic impact of MPA on the differences in cell numbers and activation for all major peripheral B and T cell subsets (e.g., Th1, Th2, Th17) were not measured. Additional research will be needed to better understand how MPA-associated immunomodulatory pathways impact the systemic cytokine milieu, cellular subsets, cell activation, and soluble mediator pathways.

The reported rates of GI intolerance have limited the clinical utility of MMF in dogs (4). The present study was not powered, nor was it of sufficient duration to measure the rates of GI side effects. Larger, prospective studies in client-owned dogs will be needed to evaluate the degree of GI intolerance induced by MPA, and to assess whether the MR PK properties of OKV-1001b (e.g., delayed release and blunted C_max_) meaningfully improve tolerability.

In summary, data generated herein suggest that MPA plasma concentrations above 600 ng/mL achieve at least 50% inhibition of T-cell proliferation. This study also suggests that the prototype OKV-1001b MPA preparation for dogs may have clinical benefit relative to IR MMF. Future studies evaluating the performance of MR configurations that maintain MPA plasma concentrations between 600 ng/m and 1,000 ng/mL are warranted in client-owned dogs with IMHA and other immune-mediated diseases.

## Data Availability Statement

The raw data supporting the conclusions of this article will be made available by the authors, without undue reservation.

## Ethics Statement

The authors confirm that the ethical policies of the journal, as noted on the journal's author guidelines page, have been adhered to and the appropriate ethical review committee approval has been received. The authors confirm that they have adhered to international standards for the protection of animals used for scientific purposes.

## Author Contributions

MK, WA, GS, PS, and OG: hypothesis generation and experimental design. MK, SC, VN, PS, and GS: organizing and conduction the experiments. MK, WA, AF, OG, and GS: interpreting and analyzing the results. MK, WA, OG, and AF: writing and revising the manuscript. All authors was involved with the preparation of this manuscript.

## Conflict of Interest

MK, GS, WA, and PS are Okava Pharmaceuticals shareholders. OG, AJF, and SC are consultants to Okava. VN was employed by Absorption Systems LP.
