# Supplementary material for: Pharmacokinetics and Pharmacodynamics of Immediate- and Modified-Release Mycophenolic Acid Preparations in Healthy Beagle Dogs
Source: Front Vet Sci. 2021 Jan 28;7:611404. doi: 10.3389/fvets.2020.611404 (PMC7876310; doi:10.3389/fvets.2020.611404)

Supplemental Figures and Tables

Supplemental Table 1: Modified Waltham Score

Grade	Description
1	Hard, dry
2	Well-formed and does not leave a trail when picked up
3	Moist and beginning to lose form. Leaves a mark when picked up.
4	Majority of form is lost, poor consistency; viscous
5	Watery diarrhea

Supplemental Table 2: MPA plasma concentrations (ng/ml) following administration of q12h MMF (10 mg/kg).

Day	Time (hr)	Dog 1	Dog 2	Dog 3	Dog 4	Dog 5	AVG	SD
Day 1	0	0	0	0	0	0	0	ND
	0.75	1590	380	4710	3510	5040	3046	2012
	4	104	92.0	222	170	175	153	54.0
	8	210	340	428	324	305	321	78.1
Day 8	0	489	207	1490	446	99.8	546	552
	0.75	1580	2990	9030	6780	6650	5406	3043
	4	404	643	747	1700	2170	1133	761
	8	362	405	1360	778	97.8	601	489
Day 15	0	637	653	129	1180	723	664	373
	0.75	2540	4460	152	4650	3410	3042	1826
	4	329	839	554	1280	423	685	384
	8	488	667	927	288	855	645	263

Supplemental Figure 1: **Gating strategy**. The following gating strategy was used: Scatter (FSC / SSC) > Singlets (FSC-A / FSC-H) > Live (L-D / SSC) > T cells (CD45+ / CD5+) > Proliferating (CD5+ / Ki-67+) (Supplemental Figure).

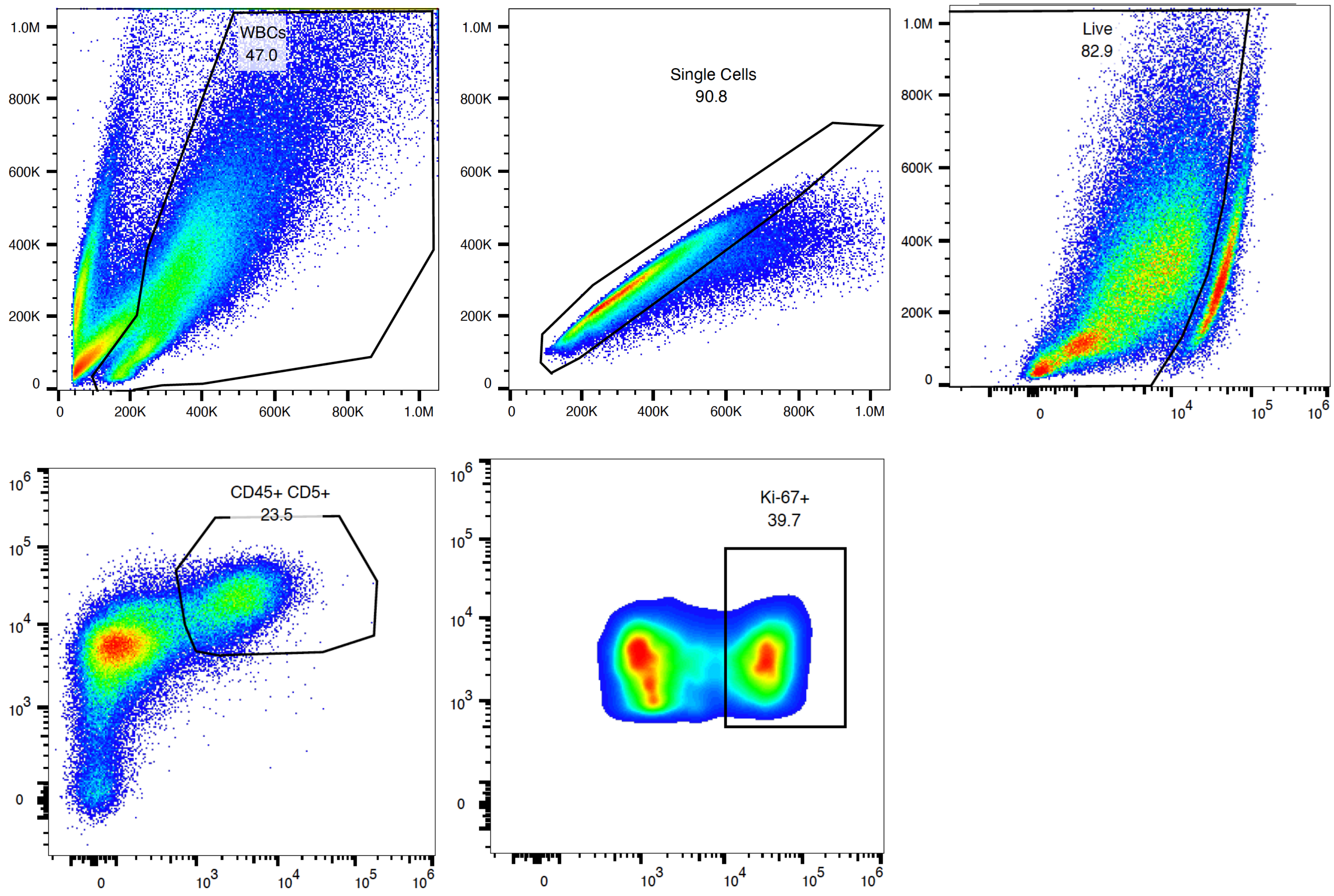

Supplemental Figure 2: Representative flow cytometry time course of MPA suppressive effects on T cell proliferation.

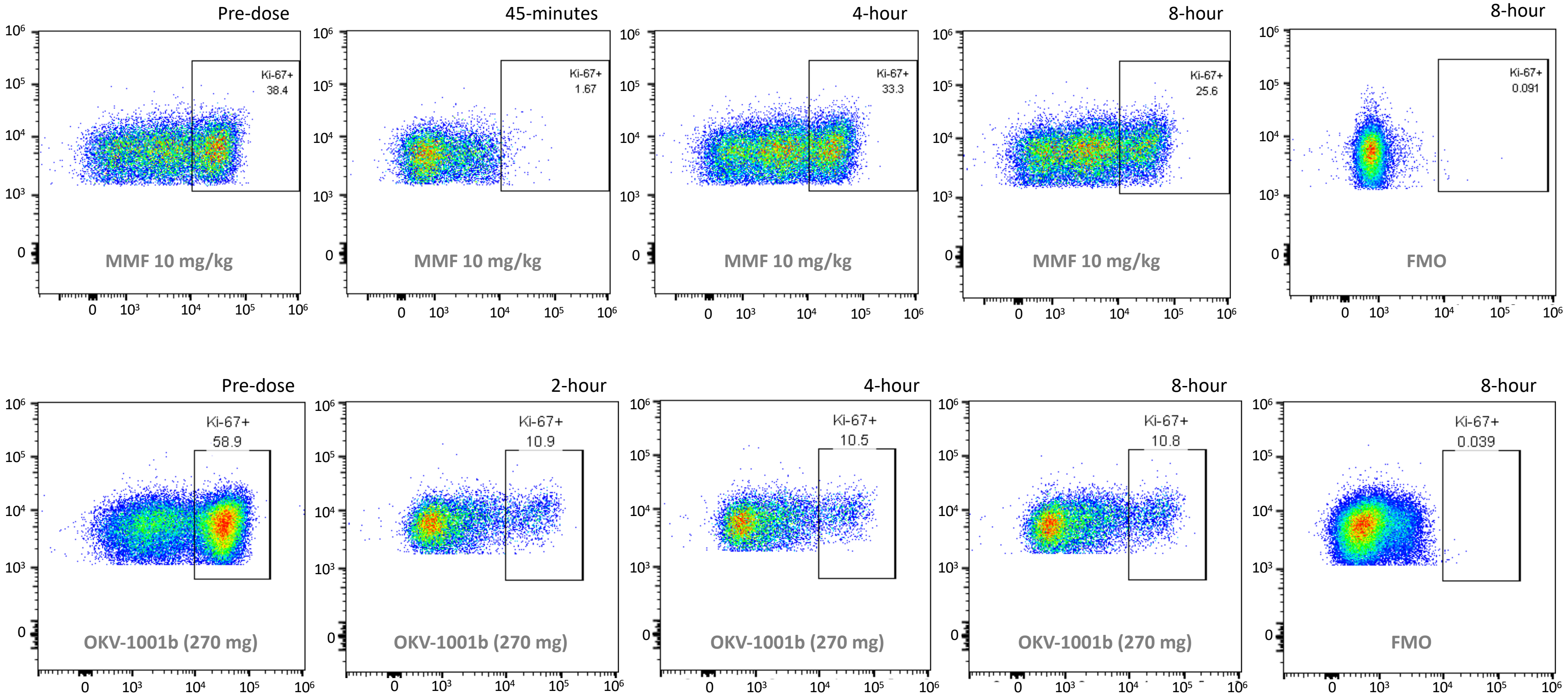

Supplemental Figure 3: ED50 graphs (Panel A = OKV-1001, 270) (Panel B= MMF)

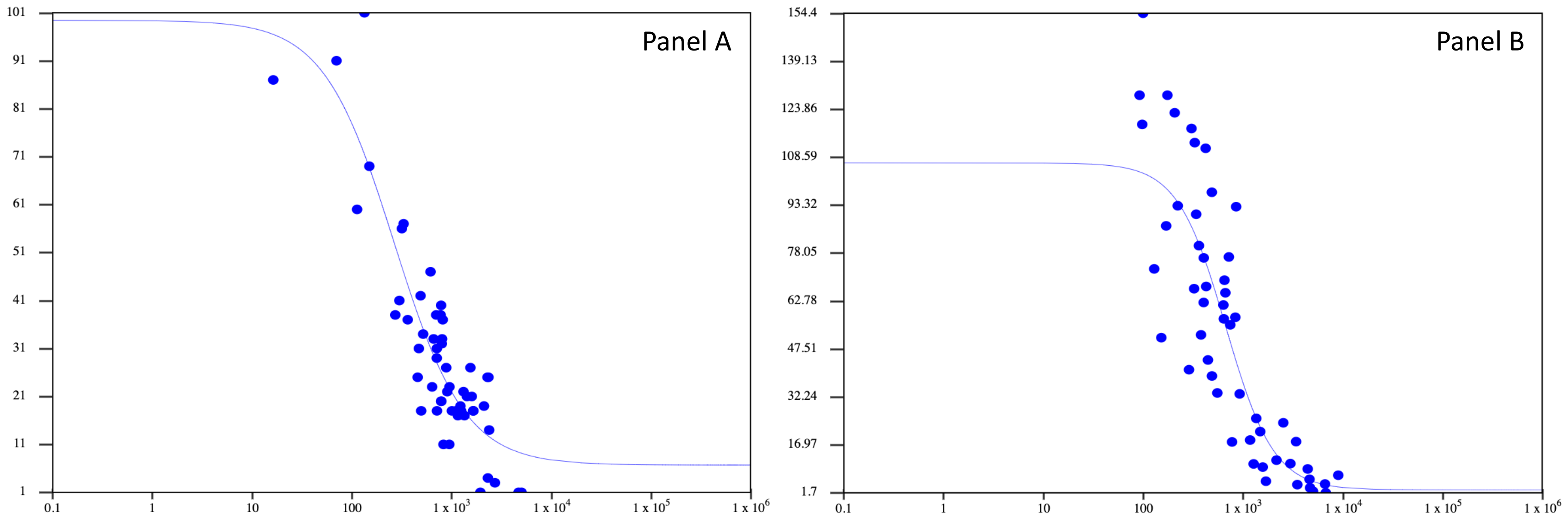

Supplement: Supplementary file 1 [file Data_Sheet_1.PDF]
