# Supplementary material for: Pharmacokinetics and Pharmacodynamics of Immediate- and Modified-Release Mycophenolic Acid Preparations in Healthy Beagle Dogs
Source: Front Vet Sci. 2021 Jan 28;7:611404. doi: 10.3389/fvets.2020.611404 (PMC7876310; doi:10.3389/fvets.2020.611404)
Supplement: Supplementary file 2 [file Data_Sheet_2.PDF]

# MPA, MPAG and AcMPAG Assay Validation

## METHOD SUMMARY

A 50 µL aliquot of acidified beagle dog plasma was precipitated with 150 µL of acetonitrile containing the internal standards MPA-$d_3$, MPAG-$d_3$, and AcMPAG-$d_3$. After vortexing and centrifugation, the resulting supernatant was transferred to a clean 96-well plate and capped for analysis. The chromatography was performed on a Waters Acquity UPLC HSS T3, 2.1 × 50 mm column and analyzed by electrospray mass spectrometry in the positive ion mode. A gradient program was used to elute the analytes using 4 mM ammonium formate as mobile phase A (MPA) and 4 mM ammonium formate in 90:10 acetonitrile:water as mobile phase B (MPB). With a flow rate of 0.300 mL/min, each analyte and their respective internal standards co-eluted with retention times of approximately 2.72 minutes for MPA/MPA-$d_3$, 1.59 minutes for MPAG/MPAG-$d_3$, and 1.87 minutes for AcMPAG/AcMPAG-$d_3$. The isobaric glucuronide metabolites are baseline resolved. Total run time is approximately 4.25 minutes. The range of the assay is 1.00-1000 ng/mL for each analyte using a 50 µL plasma sample volume.

### Calibration Curves

Calibration standards, blanks, and blanks with IS were prepared and analyzed in duplicate for each analytical run. Calibration standards were prepared fresh for each analytical batch in acidified beagle dog plasma from spiking solutions prepared in 50:50 (v:v) acetonitrile:water. Calibration curves were obtained using a quadratic regression with $1/x^2$ weighting using peak area ratios. A representative calibration curve for each analyte is shown in Figure A1

### Accuracy and Precision (A/P)

The intra-assay A/P was determined from core validation runs. These runs each contained six replicate QC samples at four concentrations including the LLOQ. Accuracy was determined by comparison of the mean measured concentrations with the nominal concentrations of these QC samples. Precision is determined as the coefficient of variation (% CV) of these replicate QC samples. The inter- and intra-assay statistics are presented in Tables A1, A2, and A3 for MPA, MPAG, and AcMPAG, respectively. The acceptance criteria were 100 ± 15.0% and 100 ± 20.0% at LLOQ. The results met acceptance criteria for all analytes.

### Stability QC Verification (Day 0)

The stability samples prepared for use with Short-Term Stability, Freeze-Thaw Stability, and Long-Term Stability assessments were analyzed on Day 0 (date of preparation) to ensure that they were correctly prepared. Stability samples were prepared at the Low QC

and High QC concentrations. The concentrations of each analyte were determined and verified in n=6 replicates on Day 0. The acceptance criteria was 100 ± 15.0% for both accuracy and precision. The results met acceptance criteria for MPA, MPAG, and AcMPAG.

.

**Short-Term (Bench-Top) Stability (STS)**

The stability of the analytes in acidified beagle plasma at room temperature while remaining on the laboratory bench was evaluated for 8 hours. The acceptance criteria was 100 ± 15.0% for both accuracy and precision. These results met the acceptance criteria for accuracy and precision of stability samples for MPA, MPAG, and AcMPAG.

**Freeze/Thaw Stability (FTS)**

The stability of the analytes in acidified beagle plasma when subjected to 3 freeze/thaw cycles was determined. FTS samples were frozen for at least one 24-hour cycle and two shorter cycles (frozen at least 12 hours each) at approximately -20°C. Thawing cycles were unassisted for approximately one hour. The acceptance criteria was 100 ± 15.0% for both accuracy and precision. These results met the acceptance criteria for accuracy and precision of stability samples for MPA, MPAG, and AcMPAG.

**Long-Term Stability (LTS)**

The stability of the analytes in acidified beagle plasma at -20°C was determined after storage for 21 days. The acceptance criteria was 100 ± 15.0% for both accuracy and precision. These data met the acceptance criteria for accuracy and precision of stability for MPA, MPAG, and AcMPAG.

Figure 1A:  Standard Curves for MPA, MPAG and AcMPAG

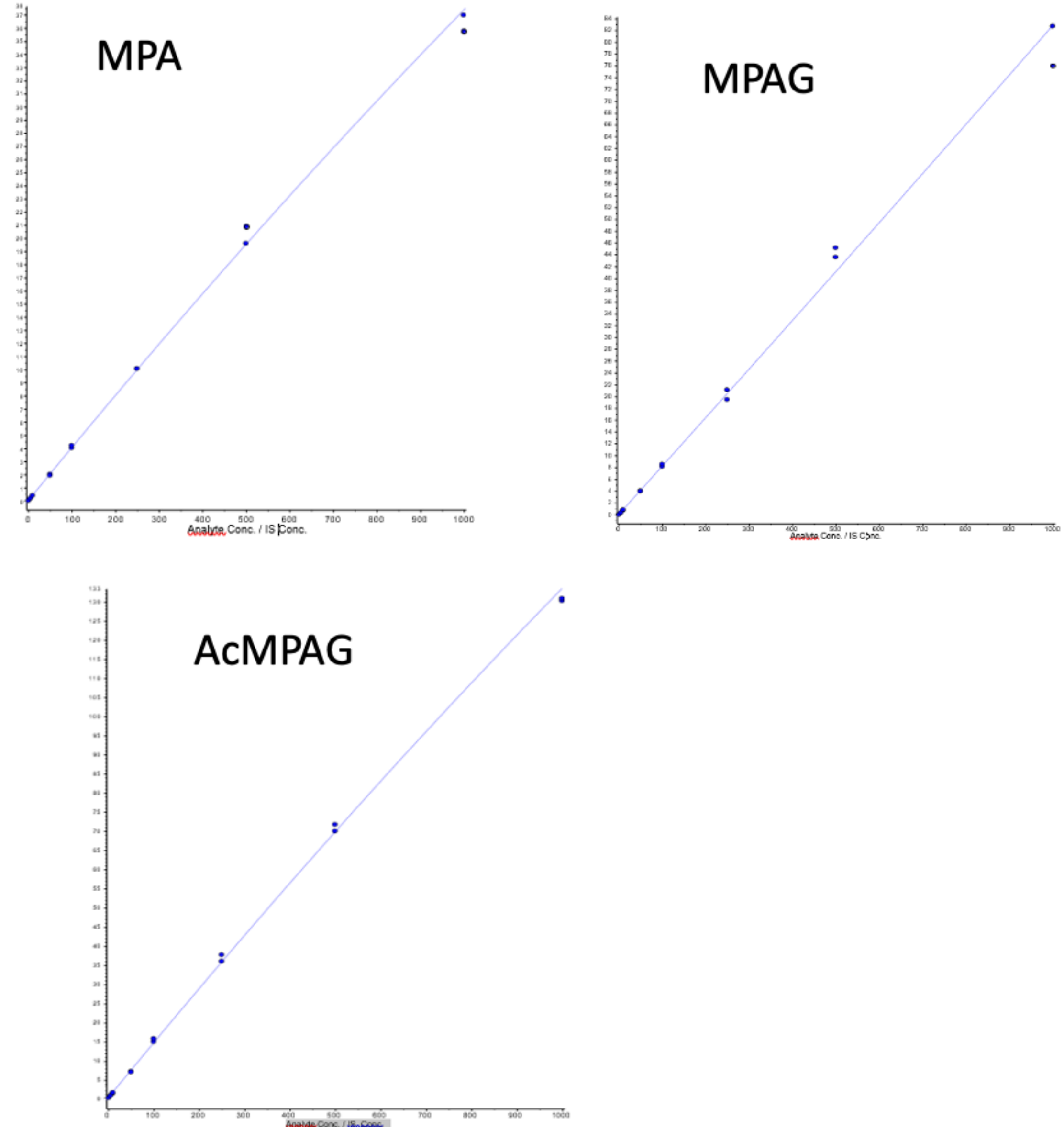

**Table A1. Intra-Assay and Inter-Assay Accuracy and Precision for MPA**

| | LLOQ QC 1.00 ng/mL | Low QC 3.00 ng/mL | Mid QC 75.0 ng/mL | High QC 750 ng/mL |
|---|---|---|---|---|
| Intra-run Mean | 1.03 | 2.98 | 75.1 | 768 |
| Intra-run SD | 0.0480 | 0.107 | 1.25 | 16.3 |
| Intra-run %CV | 4.64 | 3.59 | 1.67 | 2.12 |
| Intra-run %Accuracy | 103 | 99.2 | 100 | 102 |
| n | 6 | 6 | 6 | 6 |

**Table A2. Intra-Assay and Inter-Assay Accuracy and Precision for MPAG**

| | LLOQ QC 1.00 ng/mL | Low QC 3.00 ng/mL | Mid QC 75.0 ng/mL | High QC 750 ng/mL |
|---|---|---|---|---|
| Intra-run Mean | 0.959 | 3.08 | 75.0 | 782 |
| Intra-run SD | 0.0538 | 0.102 | 1.71 | 36.1 |
| Intra-run %CV | 5.61 | 3.30 | 2.28 | 4.62 |
| Intra-run %Accuracy | 95.9 | 103 | 100 | 104 |
| n | 6 | 5 | 6 | 6 |

**Table A3. Intra-Assay and Inter-Assay Accuracy and Precision for AcMPAG**

| | Curve Number | LLOQ QC 1.00 ng/mL | Low QC 3.00 ng/mL | Mid QC 75.0 ng/mL | High 750 ng/mL |
|---|---|---|---|---|---|
| Intra-run Mean | | 0.944 | 2.84 | 72.4 | 732 |
| Intra-run SD | | 0.0867 | 0.161 | 2.65 | 36.0 |
| Intra-run %CV | | 9.19 | 5.68 | 3.66 | 4.91 |
| Intra-run %Accuracy | | 94.4 | 94.7 | 96.6 | 97.6 |
| n | | 5 | 6 | 6 | 6 |
